# Etiology and antimicrobial susceptibility of udder pathogens from cases of subclinical mastitis in dairy cows in Sweden

**DOI:** 10.1186/1751-0147-53-36

**Published:** 2011-06-08

**Authors:** Ylva Persson, Ann-Kristin J Nyman, Ulrika Grönlund-Andersson

**Affiliations:** 1 Department of animal health and antimicrobial strategies, National Veterinary Institute, SE-751 89 Uppsala, Sweden; 2 Swedish Dairy Association, c/o National Veterinary Institute, SE-751 89 Uppsala, Sweden

## Abstract

**Background:**

A nationwide survey on the microbial etiology of cases of subclinical mastitis in dairy cows was carried out on dairy farms in Sweden. The aim was to investigate the microbial panorama and the occurrence of antimicrobial resistance. Moreover, differences between newly infected cows and chronically infected cows were investigated.

**Methods:**

In total, 583 quarter milk samples were collected from 583 dairy cows at 226 dairy farms from February 2008 to February 2009. The quarter milk samples were bacteriological investigated and scored using the California Mastitis Test. Staphylococci were tested for betalactamase production and presence of resistance was evaluated in all specific udder pathogens. Differences between newly infected cows and chronically infected cows were statistically investigated using logistic regression analysis.

**Results:**

The most common isolates of 590 bacteriological diagnoses were *Staphylococcus (S) aureus *(19%) and coagulase-negative staphylococci (CNS; 16%) followed by *Streptococcus (Str) dysgalactiae *(9%), *Str. uberis *(8%), *Escherichia (E.) coli *(2.9%), and *Streptococcus *spp. (1.9%). Samples with no growth or contamination constituted 22% and 18% of the diagnoses, respectively. The distribution of the most commonly isolated bacteria considering only bacteriological positive samples were: *S. aureus *- 31%, CNS - 27%, *Str. dysgalactiae *- 15%, *Str. uberis *- 14%, *E. coli *- 4.8%, and *Streptococcus *spp. - 3.1%. There was an increased risk of finding *S. aureus, Str. uberis *or *Str. dysgalactiae *in milk samples from chronically infected cows compared to findings in milk samples from newly infected cows. Four percent of the *S. aureus *isolates and 35% of the CNS isolates were resistant to penicillin G. Overall, resistance to other antimicrobials than penicillin G was uncommon.

**Conclusions:**

*Staphylococcus aureus *and CNS were the most frequently isolated pathogens and resistance to antimicrobials was rare.

## Background

Mastitis is the most prevalent and most costly production disease in dairy herds worldwide [1]. The most frequently isolated micro-organisms are staphylococci, streptococci and coliforms, but other micro-organisms may infect the udder. The panorama of udder pathogens varies between countries and also between types of mastitis, e.g. clinical and subclinical.

National surveys on microbial etiology of subclinical bovine mastitis have, until now, not been performed in Sweden. However, a nationwide survey on the microbial etiology of clinical mastitis was performed in 2002-2003 and revealed that *Staphylococcus (S.) aureus*, *Escherichia (E.) coli *and streptococci were the dominating findings [2]. There are no reliable data on the incidence of subclinical mastitis in Sweden, but the annual geometric average bulk-milk somatic cell counts (BMSCC), which reflects cases of subclinical mastitis in a herd, was 190 000 cells/ml in 2009 (Swedish Dairy Association, 2009). This indicates that the incidence should be rather low since a high incidence would be reflected by higher national BMSCC. Subclinical mastitis can cause substantial economic loss due to reduced milk production [3] and dairy plant fines because of high BMSCC. Moreover, cows with subclinical mastitis should be considered as a risk for spread of mastitis pathogens within and between herds and are as such of national concern.

Antimicrobials are an important tool in mastitis control programs. Therefore, surveillance of antimicrobial resistance is important to ensure optimal results of antimicrobial use and minimize the risk for selection and spread of antimicrobial resistance. The Swedish recommended antimicrobial treatment for subclinical mastitis is selected intramammary treatment at drying off (new Swedish policy for antibiotic treatment of cattle, unpublished 2011). Antibiotic treatment during lactation is not recommended according to Swedish policy in cases of subclinical mastitis. Moreover, in Sweden use of antimicrobials is on prescription only. The most recent nationwide survey of antimicrobial susceptibility was published in 2009, where the overall resistance was low [4]. This study was on clinical mastitis and a survey on antimicrobial susceptibility among pathogens causing subclinical mastitis was therefore important.

The purpose of this survey was to investigate the microbial panorama associated with subclinical mastitis and to determine antimicrobial susceptibility of udder pathogens in a random selection of dairy herds in Sweden. Moreover, a specific aim of the study was also to investigate differences between newly infected cows and chronically infected cows.

## Methods

### Study Design

A target sample size of 1000 cows in both category (newly infected or chronically infected) was set and that number should be reached during one year excluding the summer months (June, July and August). The number of cows and the number of bovine practitioners in each Swedish county was accessible and a county proportion in relation to the total number of cows in Sweden was calculated. Then, the number of cows per month to be included in the study was calculated for each veterinarian, based on the goal of 1000 cows.

On the sampling occasion the veterinarian also registered data about the cow and the herd by using a specified questionnaire; breed of the cow, lactation number, date of latest calving, milk yield at latest monthly milk recording and presence of teat lesions were recorded as were number of cows in the herd and if automatic milking systems was used.

### Animals

The two different categories of subclinical, i.e. newly infected cows (category 1) and cows chronically infected (category 2) were defined according to history in somatic cell count (SCC). Cows with a SCC ≥ 200 000 cells/ml at the latest monthly test milking and with a SCC <100 000 cells/ml at the previous test milking were classified as category 1 cows. Cows with a SCC>300 000 cells/ml at both the latest and previous monthly test milking were classified as category 2 cows. In addition, other inclusion criteria for both categories were no clinical signs of mastitis i.e. no fever, no inappetence, normal milk appearance and no consistency changes in the udder [5].

One cow from each category was sampled per herd. The same herd was allowed to be included several times but not the same cows. Milk samples were taken by veterinarians when they visited the farm for another reason. If there were more than one cow that fitted the criteria for a category, the veterinarian had been given a randomly picked digit (0 to 9) on the submission form. If one of the cows that fitted in the study had that digit on her identity tags, she was sampled. If none of the cows that fitted the criteria had that digit, the veterinarian was asked to sample the cow that had the nearest higher digit compared to the one given on the submission form.

### Milk Samples

Udder quarter milk from the two selected cows, one category 1 and one category 2, were analyzed by California Mastitis Test (CMT) [6] by the veterinarian. The CMT-reaction was graded from 1 to 5. The scores are ranked according to an increase in viscosity, where the highest viscosity (CMT 5) is more or less correlated to the highest SCC. If the udder quarter milk had CMT ≥ 3, an aseptic milk sample was taken. If more than one quarter had CMT ≥ 3 they were all sampled and then one of the samples was randomly picked at the laboratory and included in the study.

### Bacteriological Analyses

Milk samples were sent by post to the National Veterinary Institute, Uppsala, Sweden. Milk samples (10 μl) were cultured on blood (5% bovine blood) agar plates, incubated at 37°C for 16-24 h. Growth on the plates was confirmed by additional laboratory tests in accordance with the routines at the laboratory. *S. aureus *was identified by means of typical colony morphology, α- and β-hemolysis, or by coagulase reaction (coagulase-positive) when typical hemolysis zones were not present. Coagulase-negative staphylococci were identified by typical colony morphology and coagulase reaction, but were not further characterized for this paper. Streptococci were determined by colony morphology and CAMP-reaction, and 12 biochemical reactions (hippurate, aesculine, salicine, sorbitol, mannitol, raffinose, lactose, saccharose, inuline, trehalose, starch and glycerine) were used for typing to the species level. Enterococci were confirmed by Gram-staining and growth of typical colonies on SlaBa-plates (Slanetz &Bartley Medium, Oxoid Ltd., Basingstoke, England). Gram-negative bacteria with typical colony morphology, and positive for p-nitrophenyl-b-D-glucupyranosiduronic acid (PGUA) and indole were considered as *E. coli*. For other Gram-negative bacteria, oxidase reaction and API 20 E or API 20 NE (BioMérieux, Craponne, France) was used. *Bacillus *spp. was confirmed by colony morphology and Gram-staining. A milk sample was classified as positive if at least one colony-forming unit (CFU) of *S. aureus *or *Streptococcus (Str.) agalactiae *was isolated. For other agents, the presence of at least three CFUs was needed for positive classification.

Samples were classified as contaminated if three or more bacterial types were isolated from one milk sample and growth of a major udder pathogen was not identified. If growth of a major udder pathogen was found in combination with contaminating species and if the CMT was high, the sample would be diagnosed as positive for growth of the major udder pathogen. *Staphylococcus aureus*, CNS, *Str. uberis*, *Str. dysgalactiae, Str. agalactiae*, *E. coli *and *Klebsiella *spp. were selected for susceptibility testing.

### Susceptibility Testing

Isolates were tested for antimicrobial susceptibility by determination of minimum inhibitory concentration (MIC) using a microdilution method. Testing was performed according to recommendations from the Clinical and Laboratory Standards Institute [7] using VetMIC™ panels (National Veterinary Institute, Uppsala, Sweden) and cation adjusted Mueller-Hinton broth (Becton Dickinson, Cockeysville, USA). Antimicrobials and range of concentrations tested are given in Tables 1, 2 and 3. For testing of oxacillin susceptibility in staphylococci, 2% NaCl was added to the broth. Quality control strains, *S. aureus *ATCC 29213, *S. aureus *ATCC 25923 and *E. coli *ATCC 25922, tested in parallel with each batch of isolates, were on all occasions within acceptable ranges. All isolates of staphylococci were in addition examined for β-lactamase production by the "clover-leaf" method as described by Bryan and Godfrey [8]. Staphylococci with MIC for oxacillin >1 mg/l were examined for presence of the *mecA*-gene by PCR according to Smyth and others [9].

**Table 1:** Resistance and distribution of MIC for *Staphylococcus aureus *(n = 109) and coagulase negative staphylococci (CNS; n = 95).

			**Distribution (%) of MICs **^**a **^**(mg/l)**												
			
Substance	Species	Resistance (%)	≤0.03	0.06	0.12	0.25	0.5	1	2	4	8	16	32	64	>64
Cephalothin	*S. aureus*	0		0.9	46.7	45.0	6.4								
	CNS	-		4.2	25.3	53.7	9.5	6.3	4.2						

Chloramphenicol	*S. aureus*	0							0.9	21.1	75.0	3.0			
	CNS	0						1.1	6.3	71.6	21.2				

Ciprofloxacin	*S. aureus*	0		1.8	17.4	55.0	24.8	0.9							
	CNS	0		7.4	48.4	37.9	6.3								

Clindamycin	*S. aureus*	-				91.7	8.3								
	CNS	-				80.9	15.8	2.1						1.1	

Erythromycin	*S. aureus*	0				12.8	79.8	7.3							
	CNS	2				30.5	60.0	7.4	0			1.1		1.1	

Gentamicin	*S. aureus*	0					67.0	31.2	1.8						
	CNS	1					98.9	1.1							

Kanamycin	*S. aureus*	4				0.9	0.9	12.8	60.6	21.1		2.8	0.9		
	CNS	-				11.6	42.1	38.9	5.3	2.1					

Oxacillin	*S. aureus*	0				41.2	47.7	10.1	0.9						
	CNS	10			0.9	40.0	43.2	5.3	8.4	2.1					

Penicillin^c^	*S. aureus*	4	35.8	43.1	17.4			0.9	1.8		0.9				
	CNS	37	36.8	13.7	13.7	10.5	13.7	8.4	1.1	1.2	1.1				

Tetracycline	*S. aureus*	3					95.4	1.8	2.8						
	CNS	1					87.4	11.6						1.1	

Trimethoprim	*S. aureus*	-					3.7	12.8	61.5	18.3	3.7				
	CNS	-					9.5	25.3	31.6	10.5	12.6	8.4	1.1	1.1	

^a ^Thin vertical lines denote range of dilutions tested for each substance. MICs above the range are given as the concentration closest to the range. MICs equal to or lower than the lowest concentration tested are given as the lowest tested concentration. Bold vertical lines indicate EUCAST epidemiological cut-off values with exception of clindamycin and trimethoprim since the given cut-off value would have split- the distribution in an inappropriate way. When no cut-off value is available isolates are not classified as susceptible or resistant.

^b ^The isolates with MIC above cut-off were tested for the presence of mecA gene by PCR. None were positive.

^c ^NA = not applicable since classification susceptible or resistant was done according to beta-lactamase production.

**Table 2:** Resistance and distribution of MIC for *Streptococcus dysgalactiae *(n = 50) and *Streptococcus uberis *(n = 50).

			**Distribution (%) of MICs **^**a **^**(mg/l)**												
			
Substance	Species	Resistance (%)	≤0.03	0.06	0.12	0.25	0.5	1	2	4	8	16	32	64	>64
Cephalothin	*S. dysg*.	-		2.0	82.0	16.0									
	*S. uberis*	-		4.0	50.0	42.0	2.0	2.0							

Chloramphenicol	*S. dysg*.	-						6.0	28.0	66.0					
	*S. uberis*	-							36.0	62.0	2.0				

Ciprofloxacin	*S. dysg*.	-				10.0	48.0	40.0	2.0						
	*S. uberis*	-				4.0	54.0	40.0	2.0						

Clindamycin	*S. dysg*.	-				100									
	*S. uberis*	-				100									

Erythromycin	*S. dysg*.	-				100									
	*S. uberis*	-				100									

Gentamicin	*S. dysg*.	-						12.0	24.0	62.0	2.0				
	*S. uberis*	-							20.0	20.0	28.0	30.0	2.0		

Kanamycin	*S. dysg*.	-						2.0	4.0	4.0	4.0	12.0	68.0	6.0	
	*S. uberis*	-								6.0	6.0	18.0	20.0	50.0	

Penicillin^c^	*S. dysg*.	-	72.0	18.0	10.0										
	*S. uberis*	-	46.0	34.0	14.0	6.0									

Tetracycline	*S. dysg*.	-					4.0		14.0	70.0	8.0		2.0	2.0	
	*S. uberis*	-					86.0	2.0	4.0	4.0			4.0		

Trimethoprim	*S. dysg*.	-					14.0	36.0	46.0	4.0					
	*S. uberis*	-						20.0	64.0	14.0	2.0				

^a ^Thin vertical lines denote range of dilutions tested for each substance. MICs above the range are given as the concentration closest to the range. MICs equal to or lower than the lowest concentration tested are given as the lowest tested concentration. Bold vertical lines indicate EUCAST epidemiological cut-off values. When no cut-off value is available isolates are not classified as susceptible or resistant.

**Table 3:** Resistance and distribution of MIC for *Escherichia coli *(n = 17)

		**Distribution (%) of MIC**^**a **^**(mg/L)**																		
Substance	Resistance (%)	≤0.08	0.016	0.03	0.06	0.12	0.25	0.5	1	2	4	8	16	32	64	128	256	512	1024	>1024
Ampicillin	5.9							6.25	17.6	47.1	23.50				5.9					

Cefotaxime	0				70.6	23.5														

Ceftiofur	0						35.3	52.9	11.7											

Chloramp-henicol	0									5.9	82.4	11.8								

Ciprofloxacin	-		5.9	52.9	35.3															

Florfenicol	0										29.4	64.7	5.9							

Gentamicin	0							5.9	94.1											

Nalidixic acid	0								5.9	47.1	35.3	6.25								

Kanamycin	5.9									17.6	58.8	17.6	5.9							

Streptomycin	5.9										5.9	82.3	5.9		5.9					

Sulpha-metoxazole	-												29.4	64.7	5.9					

Tetracycline	5.9								64.7	29.4						5.9				

Trimethoprim	5.9						23.5	70.6							5.9					

^a ^Thin vertical lines denote range of dilutions tested for each substance. MICs above the range are given as the concentration closest to the range. MICs equal to or lower than the lowest concentration tested are given as the lowest tested concentration. Bold vertical lines indicate EUCAST epidemiological cut-off values with exception of ciprofloxacin. When no cut-off value is available isolates are not classified as susceptible or resistant.

Isolates were classified as susceptible or resistant based on species-specific epidemiological cut-off values issued by European Committee on Antimicrobial Susceptibility Testing (EUCAST) http://www.eucast.org. For staphylococci, the EUCAST cut-off value for clindamycin (>0.25 mg/l) and trimethoprim (>2 mg/l) would have split the distribution of MICs an inappropriate way. For the same reason, a higher cut-off value (>0.06 mg/l) for ciprofloxacin than recommended by EUCAST (>0.03 mg/l) was used for *E. coli*. Classification of staphylococci as resistant to penicillin or oxacillin was based on production of β-lactamase and presence of *mecA *gene respectively. Isolates were not classified as susceptible or resistant when cut-off values from EUCAST were not available.

### Statistical Analyses

Associations between the dependent variable, being a newly infected or chronically infected cow, and each of the independent factors; days in milk (DIM), breed (Swedish Red (SR), Swedish Holstein (SH) or other breed or crossbred), parity and bacteriological findings were investigated using multivariable logistic regression analysis. Herd was not included as a random factor due to too few observations per herd. However, the "cluster" command in Stata (Stata Corp., 2009; Stata Statistical Software: Release 11.1; College Station, TX, USA: StataCorp LP) was used making the standard errors allow for intragroup correlation. Before bacteriological diagnosis was included in the multivariable logistic regression analysis it was amalgamated into eight categories due to few findings of certain bacteria. The eight categories were: no bacterial finding, *S. aureus*, CNS, *Str. uberis*, *Str. dysgalactiae*, *E. coli*, other bacteria, and contaminated samples. Days in milk was centred (DIM - mean DIM) and squared to get a linear relationship with the outcome on the logit scale.

## Results

### Descriptive Data

In total, 583 cows, from 226 herds (2 - 13 cows/herd), fulfilling the selection criteria (CMT ≥ 3, and being a cow of category 1 or 2) contributed with 583 quarter milk samples. The participating herd distribution was proportionally equal to the distribution of dairy herds in Sweden (Table 4). The arithmetic mean herd size of these herds was 92 cows (50% central range (CR): 45 - 117 cows, n = 223) and the majority of farms (83%, n = 222) had conventional milking systems (parlor or tie-stall systems) while 17% had robotic milking or a combination of robotic milking and conventional milking on the farm. A total of 103 veterinarians participated in the study visiting on average 4 (range 1-11) farms each.

**Table 4:** Distribution of sampled herds per county/ies and overall distribution of dairy herds per county/ies in Sweden.

County	Number of sampled herds	Number of dairy herds (SJV; 2008)
**Skåne**	23 (9%)	657 (9%)
**Halland**	20 (9%)	413 (6%)
**Kalmar, Gotland, Blekinge and Kronoberg**	33 (14%)	1331 (19%)
**Östergötland, Jönköping, and parts of Västra Götaland county**	66 (28%)	2068 (29%)
**Västra Götaland**	8 (3%)	294 (4%)
**Stockholm, Uppsala, Södermanland, Värmland, Örebro**,	71 (31%)	1803 (25%)
**Västmanland, Dalarna, Gävleborg, Västernorrland, and Jämtland**		
**Västerbotten and Norrbotten**	11 (5%)	530 (7%)

**Total number of herds**	**232**	**7096**

The cows were mainly of the SH (53%, n = 571) or the SR (42%) breed, the rest were cross-breeds or of other breeds. Cows were of parity 1-9 (n = 579); 32% first parity cows, 25% second parity cows, 21% third parity cows and 21% of parity four or higher. Mean daily milk yield (on the test-day closest before sampling) were 32 kg of milk (50% CR: 25 - 37.5 kg of milk) and the cows were sampled on average on day 176 DIM (50% CR: 92 - 244 DIM).

### Distribution of Udder Pathogens

At least one microbial species supposedly associated with mastitis was isolated from 350 (60%) of the 583 quarter milk samples. From the majority of those quarter milk samples one species was isolated (343 of the samples (99%)). In total, 590 microbial diagnoses were obtained from 583 quarter milk samples. The distribution of microbial diagnoses is shown in Table 5. The distribution of the most commonly isolated bacteria considering only bacteriological positive samples were: *S. aureus *- 31%, CNS - 27%, *Str. dysgalactiae *- 15%, *Str. uberis *- 14%, *E. coli *- 4.8%, and *Str*. spp. - 3.1%.

**Table 5:** Distribution of bacteriological diagnoses from quarter milk samples from cows newly or chronically infected with subclinical mastitis.

Diagnosis	Newly infected cows (n, %)	Chronically infected cows (n, %)	Total
***Staphylococcus aureus***	44 (15%)	66 (22%)	110 (18.6%)
***Coagulase-negative staphylococci***	51 (18%)	46 (15%)	97 (16.4%)
***Streptococcus dysgalactiae***	21 (7.3%)	33 (11%)	54 (9.2%)
***Streptococcus uberis***	18 (6.4%)	31 (10%)	49 (8.3%)
***Streptococcus agalactiae***	0	1 (0.3%)	1 (0.2%)
**Other streptococci**	4 (1.4%)	7 (2.3%)	11 (1.9%)
**Enterococci**	1 (0.3%)	4 (1.3%)	5 (0.8%)
***Arcanobacterium pyogenes***	1 (0.3%)	1 (0.3%)	2 (0.3%)
***Escherichia coli***	7 (2.5%)	10 (3.3%)	17 (2.9%)
***Klebsiella *spp**.	3 (1.1%)	2 (0.7%)	5 (0.8%)
**Other coliform bacteria**	2 (0.7%)	1 (0.3%)	3 (0.5%)
**Other bacteria**	2 (0.7%)	1 (0.3%)	3 (0.5%)
**Contaminated**	60 (21%)	45 (15%)	105 (17.8%)
**No growth**	72 (25%)	56 (19%)	128 (21.7%)
**Total**	286	304	590

### Newly infected versus Chronic cases

The result of the multivariable analysis is presented in Table 6. The odds of finding *S. aureus*, *Str. dysgalactiae *or *Str. uberis *in milk samples was 2, 2.8, and 2.3 times higher, respectively, in milk samples from chronically infected cows compared to newly infected cows. Moreover, there was an association between being a chronically infected cow and time of sampling in relation to calving (P < 0.001). At an increased DIM at sampling from median DIM (157 DIM) to third quartile (242.5 DIM) the odds was 1.6 that the cow sampled was a chronically infected cow compared to being a newly infected cow. No other significant differences were seen between the two groups of cows.

**Table 6:** Results from the multivariable logistic regression analysis of factors associated with being a cow newly or chronically infected with subclinical mastitis in 578 dairy cows from 262 Swedish dairy herds (r2 = 00.6)

Variable	*β*	SE(*β*)	OR^a^	95% CI^b^ (OR^a^)	*P*-value
**Intercept**	-0.17	0.17	-	-	-
**Bacteriological finding**					
**1: No bacterial finding**	Ref.^c^	-	-	-	-
**2: *Staphylococcus aureus***	0.71	0.27	2.03	(1.17; 3.52)	0.01
**3: *Coagulase-negative***	0.17	0.27	1.18	(0.68; 2.06)	0.55
***staphylococci***					
**4: *Streptococcus***	0.82	0.33	2.27	(1.12; 4.60)	0.02
***dysgalactiae***					
**5: *Streptococcus uberis***	1.02	0.35	2.78	(1.38; 5.62)	0.004
**6: *Escherichia coli***	0.67	0.58	1.95	(0.68, 5.62)	0.21
**7: Other bacteria**	0.63	0.38	1.87	(0.88; 4.00)	0.11
**8: Contaminated**	0.002	0.27	1.00	(0.58; 1.72)	0.99
**Days in milk (centered)**	0.005	0.0009	n.a.^d^	n.a.^d^	n.a.^d^
**Days in milk (squared)**	-0.00001	0.000004	1.56^e^	1.33; 1.83	0.006

^a^OR = odds ratio

^b^CI = confidence interval

^c^Ref. = reference category

^d^n.a. = not applicable

^e^Odds ratio is calculated for an interquartile increase from the mean in days in milk (85.5 DIM) in both DIM (centered) and DIM (squared)

### Antimicrobial Susceptibility Testing

Of the *S. aureus *resistant to penicillin through β-lactamase production (3.7%), all had MICs for penicillin >0.5 mg/l. Two of these *S. aureus *were isolated from cows in the same herd. Distributions of MICs for the different substances are shown in Table 1.

Of the 35 CNS isolates resistant to penicillin through β-lactamase production 31 had MICs >0.12 mg/l for penicillin. The prevalence of β-lactamase producing CNS was equal between newly and chronically infected cows. Four CNS positive for β-lactamase production had MICs of 0.12 mg/l or lower for penicillin. Three CNS isolates had MICs of 0.25, 0.25 and 1 mg/l for penicillin, respectively, although they were negative for β-lactamase production. Two (2.1%) isolates tested were resistant to one or more antimicrobial; one isolate was resistant to penicillin and erythromycin and the other was resistant penicillin, erythromycin and tetracycline. One penicillin susceptible CNS isolate was resistant to gentamicin. Ten CNS isolates with MIC >1 mg/l for oxacillin were tested for presence of the *mec*A-gene by PCR but none were positive. Distributions of MICs for the different substances are shown in Table 1.

The results for streptococci are difficult to evaluate since EUCAST cut-off values are lacking for the species tested. Bimodal distributions of MICs for tetracycline for both *Str. dysgalactiae *and *Str. uberis *indicate acquired resistance in some isolates (Table 2).

Most *E. coli *were susceptible to antimicrobials tested with exception of two isolates; one was resistant to trimethoprim and the other was resistant to ampicillin, streptomycin and tetracycline (Table 3). Resistance to cefotaxime did not occur in *E. coli *and *Klebsiella *spp. indicating that none of the isolates produced extended spectrum betalactamases (ESBL).

## Discussion

This is the first national survey of subclinical mastitis in dairy cows in Sweden. The cows were sampled under strict inclusion criteria and originated from all parts of the country, why the isolates should represent a random sample of pathogens causing subclinical mastitis in Swedish dairy cows.

In this study, the most frequently isolated bacterial species was *S. aureus *followed by CNS. In most studies, staphylococci and streptococci are the most common findings in subclinical mastitis [10-16]. In a Finnish study, CNS was the most commonly isolated bacterial group (49.6% of the positive findings), followed by *Corynebacterium bovis *(34.4%) and *S. aureus *(10.2%) [17]. *Corynebacterium bovis *was uncommon in this study and is seldom recognized as a mastitis pathogen in Sweden.

One fifth of the samples submitted for culturing appeared to be negative. A reason for this may be that the number of colony forming units in milk is below the detection limit of the assay. Another reason for the negative results may be spontaneous bacteriological recovery. However, a single milk sample has been shown to have a rather low sensitivity in finding infecting bacteria [18], and if this study should be repeated the proportion of negative samples could be reduced if each cow was sampled repeatedly within a few days.

The reasons for the pre-dominance of *S. aureus *and CNS in subclinical mastitis in Sweden are not clear, but housing system and herd size are factors that might be of importance. Tie stalls are still common in Sweden and Ericsson Unnerstad *et al. *[2] reported an association between tie stalls and *S. aureus *in their study. Historically in Sweden, *S. aureus *has been the most predominant bacteria causing both clinical and subclinical mastitis. Hence, the result in this study was expected. Moreover, *Str. agalactiae*, a common finding in many other studies was very rare in this study with less than 1% of the positive findings. The reason for this could be awareness among Swedish veterinarians regarding this bacteria and effective treatment and eradication schemes.

We also found that *S. aureus*, *Str. dysgalactiae *and *Str. uberis *were more prevalent in chronic than in new subclinical cases. The reason for this is not clear, but at least *S. aureus *and *Str. uberis *are often involved in chronic cases of mastitis.

β-lactamase production is the most common resistance mechanism in staphylococci. Overall, such production was most prevalent among CNS isolates, while among *S. aureus *isolates it was lower than expected. Usually cows with β-lactamase producing *S. aureus *are culled when diagnosed in Swedish dairy herds, and that is the most plausible explanation to the low prevalence. The proportion of β-lactamase producing CNS isolates (37%) was similar to those reported from subclinical mastitis or IMI in Finland (32%), Norway (36%) and Netherlands (37%) [17,19,20]. Compared to CNS isolated from acute clinical mastitis in Sweden [4], the proportion β-lactamase producing was higher.

Overall, resistance to other antimicrobials than penicillin G was rare in all bacteria isolated from cows with subclinical mastitis and was markedly lower than in other studies [11,17]. In addition, no meticillin resistant *S. aureus *(MRSA) or CNS was found and no ESBL-producing bacteria were detected.

As management and milking systems are continuously changing in the Swedish dairy industry, changes in microbial and resistance patterns might occur in the future. Therefore, studies like the present should be repeated regularly to update the knowledge of trends in the panorama of microorganisms causing subclinical mastitis.

## Conclusions

*Staphylococcus aureus *and CNS were the most frequently isolated pathogens from cows with subclinical mastitis and resistance to antimicrobials was rare.

## Competing interests

The authors declare that they have no competing interests.

## Authors' contributions

UG conceived of the study and participated in its coordination and design and helped to draft the manuscript. YP participated in the study, participated in its coordination and drafted the manuscript. AN performed the statistical analysis and helped to draft the manuscript. All authors read and approved the final manuscript.

## References

[B1] Seegers H Fourichon C Beaudeau F Production effects related to mastitis and mastitis economics in dairy cattle herds Vet Res 2003 34 5 475 491 10.1051/vetres:2003027 14556691

[B2] Ericsson Unnerstad H Lindberg A Persson Waller K Ekman T Artursson K Nilsson-Ost M Bengtsson B Microbial aetiology of acute clinical mastitis and agent-specific risk factors Vet Microbiol 2009 137 1-2 90 97 10.1016/j.vetmic.2008.12.005 19155148

[B3] Hagnestam-Nielsen C Emanuelson U Berglund B Strandberg E Relationship between somatic cell count and milk yield in different stages of lactation J Dairy Sci 2009 92 7 3124 3133 10.3168/jds.2008-1719 19528590

[B4] Bengtsson B Unnerstad HE Ekman T Artursson K Nilsson-Ost M Waller KP Antimicrobial susceptibility of udder pathogens from cases of acute clinical mastitis in dairy cows Vet Microbiol 2009 136 1-2 142 149 10.1016/j.vetmic.2008.10.024 19058930

[B5] IDF Suggested interpretation of mastitis terminology International Dairy Federation Bulletine 1999 3 26

[B6] Schalm OW Carroll EJ Jain NC Bovine mastitis 1971 Philadelphia Lea & Febiger 128 157

[B7] Performance Standards for Antimicrobial Susceptibility Testing; Sevenenth Informational Supplement CLSI document M100-S17 2001 Wayne Pennsylvania, USA: Clinical and Laboratory Standards Institute 1 153

[B8] Bryan LE Godfrey AJ Lorian V Beta-lactam antibiotics: mode of action and bacterial resistance Antibiotics in Laboratory Medicine 1991 Baltimore, USA: William & Wilkins 648

[B9] Smyth RW Kahlmeter G Olsson Liljequist B Hoffman B Methods for identifying methicillin resistancein Staphylococcus aureus J Hosp Infect 2001 48 2 103 107 10.1053/jhin.2001.0933 11428876

[B10] Gianneechini R Concha C Rivero R Delucci I Moreno Lopez J Occurrence of clinical and sub-clinical mastitis in dairy herds in the West Littoral Region in Uruguay Acta Vet Scand 2002 43 4 221 230 10.1186/1751-0147-43-221 12831175 PMC1764198

[B11] Kalmus P Aasmae B Karssin A Orro T Kask K Udder pathogens and their resistance to antimicrobial agents in dairy cows in Estonia Acta Vet Scand 2011 53 1 4. 10.1186/1751-0147-53-4 21299911 PMC3041692

[B12] Roesch M M GD Scharen W Schallibaum M Blum JW Subclinical mastitis in dairy cows in Swiss organic and conventional production systems J Dairy Res 2007 74 1 86 92 10.1017/S002202990600210X 16978453

[B13] Bradley AJ Leach KA Breen JE Green LE Green MJ Survey of the incidence and aetiology of mastitis on dairy farms in England and Wales Vet Rec 2007 160 8 253 257 10.1136/vr.160.8.253 17322356

[B14] Wilson DJ Gonzalez RN Das HH Bovine mastitis pathogens in New York and Pennsylvania: prevalence and effects on somatic cell count and milk production J Dairy Sci 1997 80 10 2592 2598 10.3168/jds.S0022-0302(97)76215-5 9361234

[B15] Piepers S De Meulemeester L de Kruif A Opsomer G Barkema HW De Vliegher S Prevalence and distribution of mastitis pathogens in subclinically infected dairy cows in Flanders, Belgium J Dairy Res 2007 74 4 478 483 17931457 10.1017/S0022029907002841

[B16] Botrel MA Haenni M Morignat E Sulpice P Madec JY Calavas D Distribution and antimicrobial resistance of clinical and subclinical mastitis pathogens in dairy cows in Rhone-Alpes, France Foodborne Pathog Dis 2010 7 5 479 487 10.1089/fpd.2009.0425 19919286

[B17] Pitkala A Haveri M Pyorala S Myllys V Honkanen-Buzalski T Bovine mastitis in Finland 2001--prevalence, distribution of bacteria, and antimicrobial resistance J Dairy Sci 2004 87 8 2433 2441 10.3168/jds.S0022-0302(04)73366-4 15328265

[B18] Dohoo IR Smith J Andersen S Kelton DF Godden S Diagnosing intramammary infections: evaluation of definitions based on a single milk sample J Dairy Sci 2011 94 1 250 261 10.3168/jds.2010-3559 21183035

[B19] Osteras O Solverod L Reksen O Milk culture results in a large Norwegian survey--effects of season, parity, days in milk, resistance, and clustering J Dairy Sci 2006 89 3 1010 1023 10.3168/jds.S0022-0302(06)72167-1 16507696

[B20] Sampimon OC Barkema HW Berends IM Sol J Lam TJ Prevalence and herd-level risk factors for intramammary infection with coagulase-negative staphylococci in Dutch dairy herds Vet Microbiol 2009 134 1-2 37 44 10.1016/j.vetmic.2008.09.010 18977613

